# Paediatric schistosomiasis: What we know and what we need to know

**DOI:** 10.1371/journal.pntd.0006144

**Published:** 2018-02-08

**Authors:** Derick N. M. Osakunor, Mark E. J. Woolhouse, Francisca Mutapi

**Affiliations:** 1 Centre for Immunity, Infection and Evolution, Institute of Immunology and Infection Research, University of Edinburgh, Ashworth Laboratories, Edinburgh, United Kingdom; 2 Centre for Immunity, Infection and Evolution, Usher Institute of Population Health Sciences and Informatics, University of Edinburgh, Ashworth Laboratories, Edinburgh, United Kingdom; 3 NIHR Global Health Research Unit Tackling Infections to Benefit Africa (TIBA), University of Edinburgh, Ashworth Laboratories, Edinburgh, United Kingdom; Swiss Tropical and Public Health Institute, SWITZERLAND

## Abstract

Schistosomiasis affects over 200 million people worldwide, most of whom are children. Research and control strategies directed at preschool-aged children (PSAC), i.e., ≤5 years old, have lagged behind those in older children and adults. With the recent WHO revision of the schistosomiasis treatment guidelines to include PSAC, and the recognition of gaps in our current knowledge on the disease and its treatment in this age group, there is now a concerted effort to address these shortcomings. Global and national schistosome control strategies are yet to include PSAC in treatment schedules. Maximum impact of schistosome treatment programmes will be realised through effective treatment of PSAC. In this review, we (i) discuss the current knowledge on the dynamics and consequences of paediatric schistosomiasis and (ii) identify knowledge and policy gaps relevant to these areas and to the successful control of schistosome infection and disease in this age group. Herein, we highlight risk factors, immune mechanisms, pathology, and optimal timing for screening, diagnosis, and treatment of paediatric schistosomiasis. We also discuss the tools required for treating schistosomiasis in PSAC and strategies for accessing them for treatment.

## Introduction

Schistosomiasis is a tropical and subtropical disease affecting communities with limited access to safe water and adequate sanitation provision [1–3]. It affects over 200 million people worldwide (90% in sub-Saharan Africa), of which a significant number (123 million) are children [3, 4]. The health impact includes poor growth and cognition in affected children [5, 6].

Despite the higher prevalence of schistosomiasis in children, preschool-aged children (PSAC), i.e., those aged ≤5 years, for a long time were considered to be at a low risk of infection [7]; and even if infected, the impact on their health was unknown or considered negligible. Operational difficulties, including obtaining parasitology samples for diagnosis, failure to detect light infections, and inadequate knowledge about risk factors in PSAC, have biased studies towards school-aged children (SAC), i.e., ≥6 years old and adults. Infection prevalence data from a number of epidemiological studies have led to the estimation that at least 50 million PSAC in Africa are infected with schistosomiasis [8], but the true global infection and disease burden remains to be quantified. This makes it difficult to make operational and economic plans for controlling schistosomiasis in PSAC. Furthermore, gaps relating to infection, disease dynamics, and treatment need addressing if we are to deliver sustainable schistosome infection and disease control in PSAC and strengthen schistosomiasis elimination programmes.

Here, we summarise the current knowledge of paediatric schistosome infection, disease dynamics, and treatment. We also identify important knowledge gaps in paediatric schistosomiasis practice.

## Epidemiology of paediatric schistosomiasis

In schistosome-endemic areas, a significant amount of the exposure to infection in PSAC is passive (i.e., use of contaminated water in the home or children being bathed/sitting in a dish of fresh water while the guardian conducts domestic chores), particularly in the youngest children. Exposure becomes more active as the children grow (e.g., accompanying caregivers to water sources for domestic chores) [9, 10]. Therefore, in the early years of infants and young children, exposure to infection is closely linked to that of the caregiver. This disassociates as children grow older, become independent, and frequently visit contaminated water sources with friends and/or older siblings.

Exposure to infection is incremental, and almost all children in high transmission areas will have been exposed to schistosome cercariae by age one [11], with infection prevalence and intensity increasing as children grow up [12]. Thus, there is a need for inclusion of PSAC in large-scale projects that map the distribution of schistosomiasis, to inform planning for drug procurement and operational strategies for including these children in national control programmes.

In addition to the lack of burden estimates of schistosome infection and disease in PSAC, there is a paucity of incidence data in this age group. Longitudinal studies tracking the incidence of schistosome infection are required to identify and quantify exposure patterns, risks, and health impacts of infection at an early age and, most importantly, to plan treatment strategies.

## Risk factors for schistosome infection

**Fig 1:**
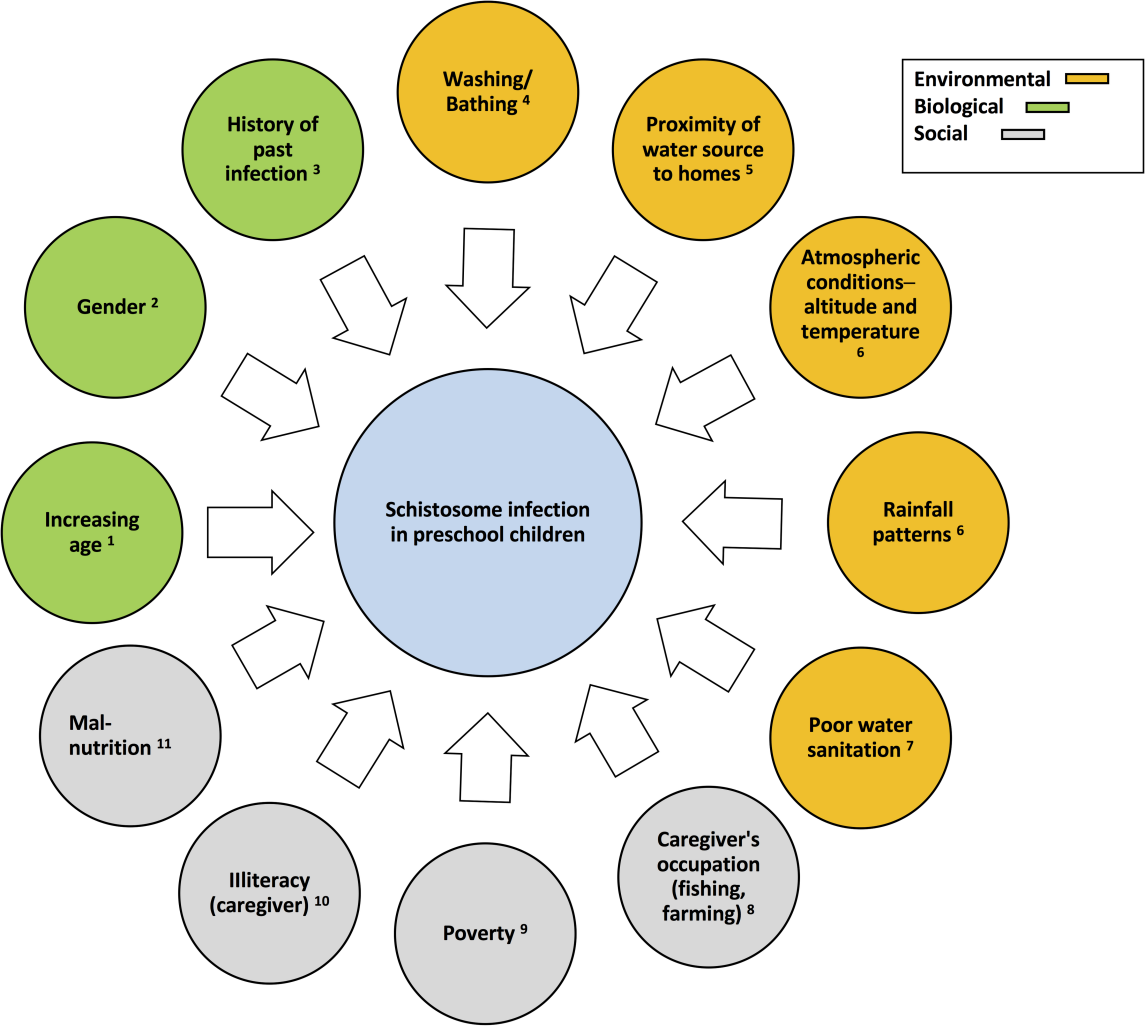
Risk factors for schistosome infection in PSAC. *Adapted from*: **1** [14]; **2** [14, 15]; **3** [16]; **4** [15], **5** [15, 17, 18]; **6** [13, 14]; 7 [19]; **8** [14]; **9** [18]; **10** [20]; **11** [21].

Several factors influence the risk for schistosome infection in PSAC, including those already identified in other age groups [Fig 1]. Environmental factors (including temperature, seasonal rainfall patterns, and altitudes) influence the survival of the intermediate host snail, as well as parasite development in the snail, affecting the force of transmission and infection [13, 14]. Exposure patterns of the human host are also affected by climatic changes (e.g., hotter seasons prompt increased recreational use of infected water sources), and passive contact with infective water can increase amongst PSAC, while they are waiting for their caregivers to complete chores. Exposure will vary in both the surface area exposed as well as the duration and frequency of exposure [15].

In addition to these well-studied risk factors, we are beginning to appreciate the impact of the human gut and urinary microbiome [22–24]. The gut microbiome is believed to be particularly variable in the early years of life [25]; for example, we have reported that the relative abundance of different bacterial taxa varies within the first three years of life [26]. The gut microbiome plays a vital role in maintaining barrier integrity, and subsequently impacts the host immune system [27], and contributes to the observed differences in infection patterns [28]. Recently, we demonstrated a significant difference in the microbiome structure (frequency and diversity of the host bacteria species) between schistosome-infected and uninfected Zimbabwean children aged 1 to 10 years [26]. From the study design, the mechanistic direction of this relationship and the influence by other factors was unclear. These aspects together with any mechanistic pathways are yet to be elucidated. In this age group, the impact of gut microbiome dysbiosis on nutritional status is of particular importance.

Emerging ideas from initiatives such as the Human Microbiome Consortium suggest that our focus on PSAC should be to understand how the microbiome functions and influences innate susceptibility to schistosome infection, if at all [29]. Helminths modulate host immune responses for their survival, and this in turn has a negative impact on the host microbiome structure and nutrition [23]. Increasing research in nutraceuticals should thus inform additional child supplementation programmes in affected countries. At present, schistosomiasis interventions targeting the microbiome remain theoretical, as mechanistic studies to establish and quantify causal relationships are lacking.

In schistosome-endemic areas, malnutrition and undernourishment are significant childhood problems. Nonetheless, there are no studies detailing the attributable fraction of malnutrition due to schistosomiasis or more importantly, the impact of treatment on these factors. This information is necessary to advocate for control strategies to prioritise PSAC. Studies have also yet to establish the role/relevance of anthropometric measures as a tool for identification of the risk of malnourishment among PSAC in schistosome-endemic areas.

## Schistosome-specific immune responses and clinical relevance

The interest in immunity in schistosome infections is twofold. First, hosts in endemic areas develop protective acquired immunity against infection/reinfection, and second, the severe and chronic clinical manifestations of schistosomiasis are immune mediated. Decades of studying immune responses in both human and experimental models have given insights into the aetiology and clinical manifestation of morbidity and immunopathology and into the development of schistosome-specific protective immune responses. The paradigms presented by these studies are not easy to extrapolate to PSAC, due to differences in the natural history of infection. Human studies have largely focused on SAC and adults with distinct histories of infection, which impacts on their immune phenotype [30]. Furthermore, most experimental studies on schistosome immunology use mouse models, which are limited in their ability to recapitulate morbidity and pathology occurring in the natural human host. For instance, eggs implanted in mice were recently used to develop an experimental model for urogenital schistosome pathology [31], but the relevance of this to the human disease forms in young children is unclear.

### Acute and chronic stages of schistosome infection

In endemic areas, the acute stage of schistosome infection—including the Katayama syndrome—receives very little research or clinical intervention compared to other areas [32, 33]. As detailed above, the first infection event in most endemic areas occurs early at the pre-school age, but to date, no studies of schistosome cercarial dermatitis in PSAC have been published. The immune responses occurring in the early stages of infection are poorly documented in humans, and these events in PSAC would be very informative on the nature and development of both pathological and parasite-protective immunity.

In studies of SAC, protective immunity against schistosomiasis is characterized by a dynamic shift in the balance between effector and regulatory (humoral and cellular) immune responses, with effector responses surpassing the regulatory responses as infection progresses and exemplified by Nausch and colleagues [34]. We have previously proposed a threshold hypothesis [35] to explain the switch from a predominantly regulatory phenotype to an effector immune phenotype as infection progresses. Nonetheless, there is a paucity of immunology studies in PSAC detailing the dynamics occurring at the transition of infection from acute to chronic. Knowledge of these responses will be informative for schistosome vaccine development and deployment, as the ideal vaccine would target all children at risk of schistosome infection who may have already experienced their first schistosome infection exposure/infection event.

### Drug-induced protective immunity

Studies in SAC and adults have shown that treatment of schistosome infections with Praziquantel (PZQ) enhances schistosome-specific immune responses by (i) removing the immuno-suppressive effects of the adult worms [35–37], and (ii) introducing large amounts of parasite-specific antigens to the immune system to reach the threshold required to induce an immune response [38–40]. These changes are associated with reduced reinfection rates [38, 41]. One of the consequences of the previous schistosome treatment guidelines was that studies on the effects of PZQ were focused more on SAC and adults. Thus, the immunological consequences of PZQ in PSAC remain poorly characterised. In a recent study in Zimbabwean PSAC, we showed an increase in anti-parasite IgM and IgE titres, six weeks after treatment with PZQ, and this was associated with resistance to reinfection [42]. This mimics the development of naturally acquired immunity, albeit (in this case) accelerated by treatment [39, 40, 43].

Evidence from experimental studies suggest that a more rational approach to the timing of helminth treatment may have additional benefits beyond the transient removal of infection and may also speed up the development of protective immunity more effectively than treating chronic infection. These studies have shown that treatment of the first helminth infection induces earlier and greater levels of protection against reinfection by preferentially inducing the effector over regulatory responses [44–46]. In this case, chronic infection induces a constant state of anti-parasite Th2 response, reducing the overall effect of Th1 effector responses required to fight infection [47]. This suggests that treatment of the first schistosome infection could have a longer-lasting impact on susceptibility to reinfection and presents the possibility of targeting treatment for maximum benefit in terms of the future health of the child. As detailed above, a single PZQ treatment can induce resistance to reinfection in PSAC [48], suggesting that if treatment of PSAC is optimally timed then repetitive treatments may be reduced or not required [49, 50]. Longitudinal studies determining the optimal treatment time are needed to inform the treatment guidelines for PSAC. Now, we have a window to conduct these studies, while there is a concerted effort to make a paediatric formulation of PZQ for deployment within the next few years.

## Pathology and morbidity

Schistosome pathology and morbidity are still being defined in older children and adults, e.g., only recently was urinary schistosomiasis renamed urogenital schistosomiasis to reflect the increased recognition of the genital manifestations of *S*. *haematobium*-related disease [51]. Pathology and morbidity remain poorly described and studied in PSAC [10] compared to SAC. However, studies are beginning to shed light on schistosome morbidity in this group, e.g., a recent study using ultrasound has contributed to describing urinary morbidity in PSAC [52].

Describing morbidity and pathology at all stages of schistosome infection in PSAC will inform overall healthcare in this age group. In PSAC, clinical symptoms upon schistosome exposure and infection (e.g., cercarial dermatitis and fever) may go unrecognised [5, 53, 54] or be mistaken for symptoms of other illnesses such as malaria, which present with similar symptoms (e.g., fever).

It is also possible that schistosome infections have wider impacts in childhood health. In PSAC, chronic prenatal exposure/sensitisation to helminth infection is reported to be associated with reduced efficacy of childhood vaccines through induction of a persistent Th2 response phenotype [55, 56]. Chronic exposure is also thought to be associated with environmental enteropathy, which affects the efficacy of vaccines at infancy [57]. Reduced vaccine efficacy means infection and disease from the vaccine-preventable infections. Thus, controlling schistosomiasis in PSAC may have the added health benefit of improving vaccine efficacy; appropriate studies are required to investigate if this would be the case.

Schistosome infections damage epithelial barriers, resulting in anaemia, poor nutrition, and growth [58, 59]. Thus, even with the low parasite burdens in PSAC, the pro-inflammatory response generated can quickly lead to chronic morbidity [60]. For example, schistosome-associated anaemia and malnutrition in older Kenyan children (5–18 years old) was attributed to pathology beginning much earlier in life [58]. The nutritional effects could also be attributed to microbiome dysbiosis as reported in the experimental mouse model of helminth infection [61]. Thus, the impact of schistosomiasis in PSAC should be an integral part of interventions, targeted at improving early child health and development.

Poor awareness, recognition, and lack of understanding and quantification of schistosome-associated morbidity has previously made schistosome control in PSAC a lower priority than in older children and adults, but concerted efforts are now beginning to correct this. There is a need for biomarkers of environmental enteropathy in PSAC [59], mechanistic studies to infer causality, and well-defined predictors of growth and nutrition in PSAC exposed to schistosomiasis.

### Paediatric schistosomiasis in coinfected hosts

Similar to all other age groups in tropical regions, PSAC are at risk of coinfections and therefore, comorbidities. Studies in older children (5–18 years old) indicate that the presence of multiple infections in endemic areas lead to significant morbidity, including anaemia and malnutrition and these are important in the formative years of PSAC [58]. Specific coinfection studies include malaria–schistosomiasis, where studies in SAC suggest that malaria severity is compounded by schistosome infection, and malaria treatment is more effective when schistosome infection is also treated [62, 63]. To date, no detailed studies have been conducted on the fraction of malaria deaths in PSAC that are attributable to schistosome coinfection. In addition, *S*. *haematobium* infection has been shown to reduce the level of protective IgG responses to malaria vaccine candidates [64]. In another instance, in vitro studies have shown that *Salmonella* can evade optimal antibiotic treatment by binding to schistosomes using fimbral proteins (FimH) present on its surface [65], and that effective treatment of schistosomiasis results in the release of *Salmonella*, causing septicaemia [66]. These findings need validation in PSAC to allow the implementation of appropriate interventions.

## Diagnosis of infection

One operational model being proposed for the treatment of schistosomiasis in PSAC is that diagnosis is made before treatment, unlike the mass drug administration (MDA) approach where SAC are treated without diagnosis [67]. This means that in the “diagnose and treat” model, accurate point of care (POC) diagnostics are key to guide the targeted treatment.

Direct parasitological methods (Kato–Katz and urine filtration) are recommended by the WHO [68] and are convenient, specific, rapid, cheap, and suitable for field applications in PSAC [69, 70]. Nonetheless, in addition to the already known limitations of low sensitivity of the parasitological methods in SAC and adults, these methods have the added operational challenge of obtaining adequate urine and stool samples in PSAC [71], particularly the collection of replicate samples on different days to improve sensitivity [72].

Various other methods have been developed in the laboratory to address the issues of sensitivity and are at different stages of translation into field tools. Immunological detection of schistosome-specific antibodies in sera has been shown to be more sensitive in diagnosing infection in PSAC than parasitological methods [12]. The non-invasive detection of worm antigens in urine, i.e., circulating anodic antigen (CAA) [73, 74] and circulating cathodic antigen (CCA) [75] have also been evaluated in PSAC. These have been shown to be a reliable tool in diagnosing *S*. *mansoni* [76] but less so in *S*. *haematobium* diagnosis [77]. Issues of cost, which pose challenges in low resource settings where schistosomiasis is endemic [78, 79], would also need to be overcome. Molecular techniques detecting parasite DNA in urine have shown promise in PSAC [80, 81], although the absence of POC versions of these tests means they require expertise and specialised equipment [82]. Compared to CCA and parasitology, polymerase chain reaction (PCR) was most effective in detecting low intensity *S*. *mansoni* and *S*. *haematobium* infections [83, 84]. The use of cell-free parasite DNA present in urine and saliva [85] in PCR techniques can ease sampling difficulties in PSAC and detect early infections [86]. MicroRNAs have been characterised in animal models of *S*. *japonicum* [87] and *S*. *mansoni* [88]. Despite conflicting reports on the utility of miR–223 as a biomarker for helminth infections [88, 89], microRNAs have great diagnostic potential in PSAC and require further exploration. However reliable these diagnostics turn out to be at the lab bench, their true utility will be tested in the field.

## Morbidity markers for schistosomiasis

The majority of morbidity biomarkers associated with schistosomiasis are non-specific, relating to physiological, biochemical, and immunological changes. As such, their interpretation in the light of coinfections and comorbidities is complex. Recent years have seen studies conducted in PSAC to evaluate the utility of morbidity markers described in SAC. These have included evaluation of the classic urine markers, haematuria and proteinuria in *S*. *haematobium* infection (e.g., in Nigeria [90] and Zimbabwe [91]), and the urine albumin-creatinine ratio (UACR) in Zimbabwe [91]. Faecal occult blood (FOB), the presence of cryptic blood in stool, results when *S*. *mansoni* eggs perforate the intestinal mucosa and cause a small release of blood into the bowel [92, 93], along with calprotectin released by granulocytes in response to the accompanying inflammation [94]. FOB and calprotectin have been evaluated in PSAC in Uganda [92, 95]. In these studies, FOB and calprotectin correlated positively with *S*. *mansoni* infection pre- and post-treatment although there may be exceptions.

The challenge with these non-specific markers particularly is to determine how schistosomiasis influences them; this is a difficult task because even determining the fraction attributable to schistosome infection does not indicate causation. Equally important is the lack of specific information on what they mean in terms of the child’s overall current and future health. There is the need for a knowledge base addressing these two issues if these morbidity markers are to form part of the diagnostic tool kit to inform the “diagnose and treat” approach for PSAC.

## Schistosome treatment in PSAC

Following the recommendation to treat PSAC infected with schistosomes, there is a need for a drug formulation that is suitable for PSAC. PZQ is the drug of choice for treating schistosomiasis at a recommended dose of 40–60 mg/kg body weight. In ill-resourced endemic areas, a height–dose pole has been developed as a surrogate to weight scales for operational purposes [96]. Modifications were made to extend the original dose pole used for SAC and adults to include PSAC [97, 98]. PZQ is currently administered to PSAC as crushed tablets with juice or bread [4, 10]. Followed by an endorsement from the WHO [99], a recent randomised dose-ranging trial reports that a single 40 mg/kg dose of PZQ can be used for treatment in PSAC [100]. Although PZQ is confirmed safe and efficacious [101], there is little information on its pharmacokinetics in PSAC [102, 103], and this is compounded by variability in bioavailability, influenced by brand [104, 105].

PSAC tolerate PZQ well with few reports of adverse effects: normally abdominal pain, vomiting, fatigue, and diarrhoea that resolve within 24 hours [98, 100, 101]. Contrary to suggestions of a higher efficacy dose (>40 mg/kg) in PSAC [106], a recent study reported on the efficacy and safety of escalating doses (20 mg/kg, 40 mg/kg, and 60 mg/kg) of PZQ in PSAC and SAC [100]. PZQ showed a flat dose-response curve in PSAC compared to that in SAC and the current 40 mg/kg remains the best option in PSAC [99]. In PSAC, PZQ can be administered with other antihelminthics (e.g., Albendazole) as a deworming package. There are currently no drug–drug interaction studies that have been conducted on these drugs in PSAC [107]. PZQ is metabolised by the cytochrome P_450_ enzymes as shown in experimental studies [108, 109]. With growing evidence of genetic diversity for cytochrome P_450_ variants in schistosome-endemic regions in Africa [110], there are implications for PZQ metabolism and treatment efficacy which remain to be elucidated in PSAC.

### Paediatric PZQ formulation

Operationally, the large size and bitter taste of the existing tablet are associated with administration difficulties in PSAC [100, 111]. To address these issues, the Paediatric Praziquantel Consortium has produced a paediatric PZQ tablet meeting a previously suggested target product profile (TPP) [4, 112]. This formulation—a smaller, orally dispersible tablet with a masked taste—has successfully undergone phase I clinical trials and phase II trials are currently underway (http://www.pediatricpraziquantelconsortium.org/).

## Accessing PSAC for schistosome treatment and control

Preventive chemotherapy programmes targeting SAC are conducted via MDA with antihelminthics (usually PZQ and Albendazole) administered in the school setting. PSAC do not go to school (although some may be in early child development centres) and the “diagnose and treat” recommendation makes it difficult to treat them outside a health setting. One strategy is to access PSAC through the Expanded Programme on Immunization (EPI) in primary health centres [4], and our studies in Zimbabwe confirm the feasibility of accessing PSAC at health centres [101]. However, in some health systems, the EPIs may already be “crowded,” implementing other interventions at the same time; hence, alternative access strategies are required. A report from a recent meeting [67] included the approach of empowering health workers to identify clinical cases (i.e., non-specific signs of suspected cases) of schistosomiasis in PSAC and treating them, as a potential means of accessing PSAC. This approach has both advantages and disadvantages; the main disadvantage is the potential of treating non-specific clinical symptoms due to infections/diseases other than schistosomiasis.

## Conclusion

There is now a global move to elevate research on paediatric schistosomiasis and to promote control, not only for child health but also to move towards eliminating schistosomiasis. The control of paediatric schistosomiasis will only be prioritised in countries with limited health budgets when (i) there is compelling evidence on the burden of infection and disease on child health and development, (ii) there are cost-effective intervention tools, and (iii) engagement at stakeholder and end-user levels. The research community can contribute to all three aspects, and bridging the knowledge gaps highlighted in this review will make a significant contribution to the control of schistosomiasis in PSAC.

**Key learning points**

- Preschool-aged children (PSAC) carry significant infection and morbidity to schistosomiasis. There is a treatment gap in mass drug administration, and studies mapping the level of infection and morbidity in this group. Infection and disease quantification is critical to inform planning and deployment of schistosome treatment in PSAC.
- Existing and new morbidity markers, along with improved multiple diagnostic tools, are useful for targeted disease identification and treatment in PSAC. The utility of these along with a proper strategy is important. There is a need for better-defined disease markers for this age group.
- Treatment with Praziquantel is safe and efficacious. Owing to existing challenges with the current formulation, there is a need for a paediatric formulation, a better dosing system, a better treatment strategy, and innovative ways of accessing PSAC for treatment.
- Most PSAC will experience infection early, and there is a need to characterise and quantify the consequences of these early infections in terms of impact on the overall health of children.
- PSAC are capable of mounting an adaptive immune response to schistosome infections, and a single treatment can illicit this response. Exploring the applicability of this in resistance to reinfection and morbidity is important.

**Top Papers**

- Wami WM, Nausch N, Midzi N, Gwisai R, Mduluza T, Woolhouse M, et al. Identifying and evaluating field indicators of urogenital schistosomiasis-related morbidity in preschool-aged children. PLoS Negl Trop Dis. 2015;9(3):e0003649. doi: 10.1371/journal.pntd.0003649. PubMed PMID: 25793584; PubMed Central PMCID: PMCPMC4368198
- Mutapi F, Rujeni N, Bourke C, Mitchell K, Appleby L, Nausch N, et al. *Schistosoma haematobium* treatment in 1–5 year old children: safety and efficacy of the antihelminthic drug praziquantel. PLoS Negl Trop Dis. 2011;5(5):e1143. doi: 10.1371/journal.pntd.0001143. PubMed PMID: 21610855; PubMed Central PMCID: PMCPMC3096601.
- Bustinduy AL, Friedman JF, Kjetland EF, Ezeamama AE, Kabatereine NB, Stothard JR, et al. Expanding Praziquantel (PZQ) Access beyond Mass Drug Administration Programs: Paving a Way Forward for a Pediatric PZQ Formulation for Schistosomiasis. PLoS Negl Trop Dis. 2016;10(9):e0004946. doi: 10.1371/journal.pntd.0004946. PubMed PMID: 27658198; PubMed Central PMCID: PMCPMC5033572.
- Rujeni N, Nausch N, Midzi N, Cowan GJ, Burchmore R, Cavanagh DR, et al. Immunological consequences of antihelminthic treatment in preschool children exposed to urogenital schistosome infection. J Trop Med. 2013;2013:283619. doi: 10.1155/2013/283619. PubMed PMID: 23840222; PubMed Central PMCID: PMCPMC3687481.
- Mutapi F. Changing policy and practice in the control of pediatric schistosomiasis. Pediatrics. 2015;135(3):536–44. doi: 10.1542/peds.2014-3189. PubMed PMID: 25687146.
